# Novel Anatomical Variation of Left Coronary Artery Origin at the Sinotubular Junction Level With Coeval Hypoplastic Left Circumflex, Superdominant Right Coronary Artery, and Obstructive Coronary Artery Disease: A Case Report

**DOI:** 10.7759/cureus.59715

**Published:** 2024-05-06

**Authors:** Parishmita Barman, Andrew John

**Affiliations:** 1 Department of Radiology, Sree Balaji Medical College & Hospital, Chennai, IND; 2 Department of Radiology, Kiran Multi Super Speciality Hospital & Research Centre, Surat, IND

**Keywords:** sinotubular junction, right coronary artery (rca), left main coronary artery disease (lmcad), left circumflex artery (lcx), left anterior descending artery, hypoplasia, computed tomography coronary angiography, coronary artery angiography, coronary artery anomalies origin

## Abstract

The isolated origin of the left coronary artery (LCA) ostium at the level of the sinotubular junction (STJ) has been described previously. Congenital absence of the left circumflex (LCx) coronary artery has also been documented with superdominant right coronary arterial circulation, either in the presence or absence of coronary artery obstruction. Earlier literature has linked the association of an absent LCx coronary artery with a superdominant right coronary artery (SRCA) but not with a hypoplastic LCx coronary artery (HLCx). The present case report details the case of a 37-year-old thin, athletic male with the risk factors of diabetes and hypertension who was admitted to the emergency unit of our hospital for losing consciousness while bicycling in the street. The current report establishes a combined association of LCA anomaly origin at STJ level along with HLCx and SRCA condition with the burden of mild to moderate coronary artery disease involving proximal left anterior descending artery, LCx, and mid right coronary artery in the literature for the first time. Further, the case report advocated that the presented case carries the risk of malignancy. Hence, with the advancement of modern imaging technologies, computed tomography angiography should be the first choice of imaging modality rather than coronary angiography to prevent fatal outcomes. Interventional cardiologists, cardiothoracic surgeons, and radiologists should have properly defined knowledge of coronary artery anatomy and associated pathology, as it is important for coronary cannulation or any coronary interventions.

## Introduction

Around 1% of healthy people have inadvertently acquired coronary artery anomalies (CAAs), which are exceptional congenital diseases [1]. An angiography’s findings were used to categorize CAA into seven distinct categories [2,3]. Nontraumatic sudden death research in adolescents has shown that 30% of CAAs are a relatively prevalent cardiac aberration among them, and the clinical outcome of these variants is largely benign [4,5]. Most afflicted were adolescents who died of sudden cardiac death irrespective of exhibiting any clinical signs, and their diagnosis was made only with an autopsy [6]. The CAA, as an anatomical manifestation of the left coronary artery (LCA) stem, varies greatly in terms of its length, level of origin, and quantity of terminating branches. Left coronary ostia would be found either below or at the sinotubular junction (STJ) level in 65% of cases and higher the border of STJ in a significant proportion of cases in 35% [7]. Another noticeable abnormality is hypoplasia of the left circumflex (LCx) coronary artery (HLCx) [8]. About 90% of patients with congenitally absent LCx arteries would have superdominant right coronary artery (SRCA) circulation, demonstrating that it is nearly always linked to the genetic impairment of LCx as a component of anatomical compensation aimed at meeting the demands of the typical territory of LCx [9]. Individuals with a double right coronary artery (RCA) emanating from a single ostium are at a significantly higher risk of developing atherosclerotic coronary artery disease (CAD). Unexpected implications of atherosclerotic CAD may arise from the unintentional discovery of a double RCA undergoing coronary angiography (CA) or cardiac surgery [10].

## Case presentation

We present the case of a 37-year-old thin, athletic male with the risk factors of diabetes and hypertension who was admitted to the emergency unit of our hospital for losing consciousness while bicycling in the street. The patient was completely alert and oriented at the time of the presentation. His family history did reveal the presence of cardiac chamber enlargements in his elder brother. He was strictly on regular antidiabetic and antihypertensive medicine. The patient’s key complaints were dyspnea, chest pain, and palpitation, and he was further evaluated. He had experienced palpitations and dyspnea before the syncopal attacks, without angina. A physical examination of his vital signs revealed no significant abnormalities. The cardiac auscultation showed normal findings. Biochemical studies exhibited normal values of blood glucose, hemoglobin, and serum electrolyte levels. His cell count ranges were also within normal limits. His ECG revealed normal sinus rhythm with slightly elevated heartbeats of 110 beats per minute and no anomalies in the ST segment or enlargement of the cardiac chamber. Nil significant chamber hypertrophy, intracardiac shunt, or evidence of valvular disease and regional wall motion abnormality were identified with mild global dysfunction of left ventricular systolic function with an ejection fraction (LVEF) of 46% in the transthoracic echocardiogram and normal right ventricular function. Given the enlargement of cardiac chambers in the family, risk factors, and clinical presentation of obstructive CAD and decreased LVEF, the patient was advised to undergo computed tomography angiography (CTA).

**Figure 1:**
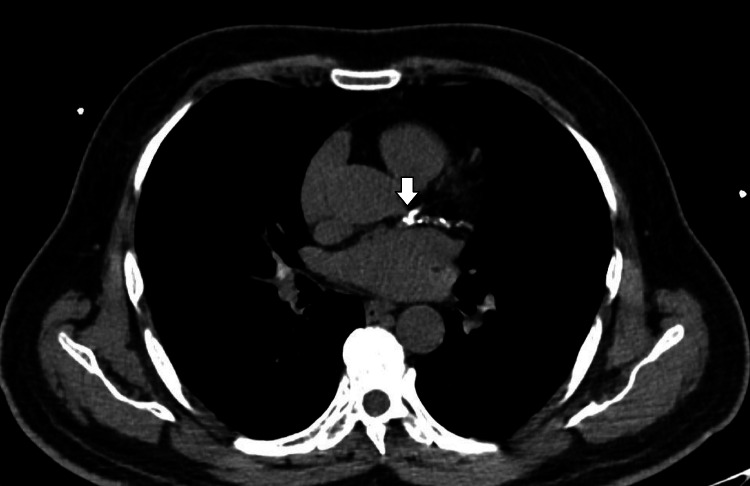
CT image of calcified LCx coronary artery The white arrow represents the lesion. LCx, left circumflex Image source: Alam et al. (2023) [10]

**Figure 2:**
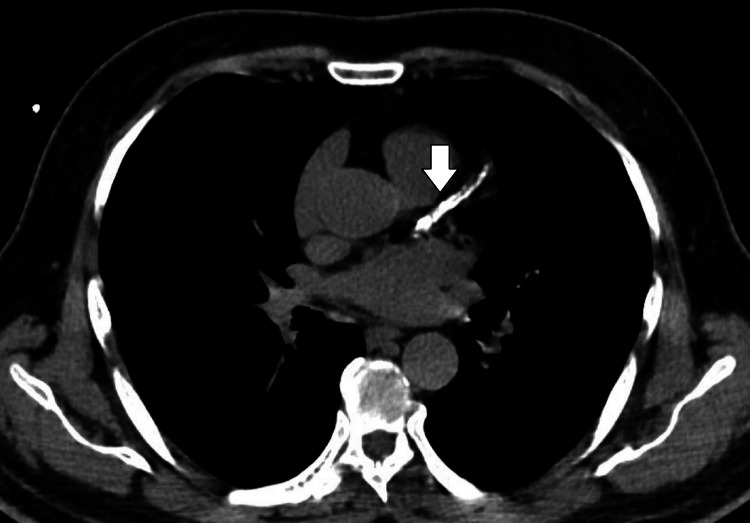
CT image of the LAD with a calcified lesion The white arrow represents the lesion. LAD, left anterior descending artery Image source: Alam et al. (2023) [10]

**Figure 3:**
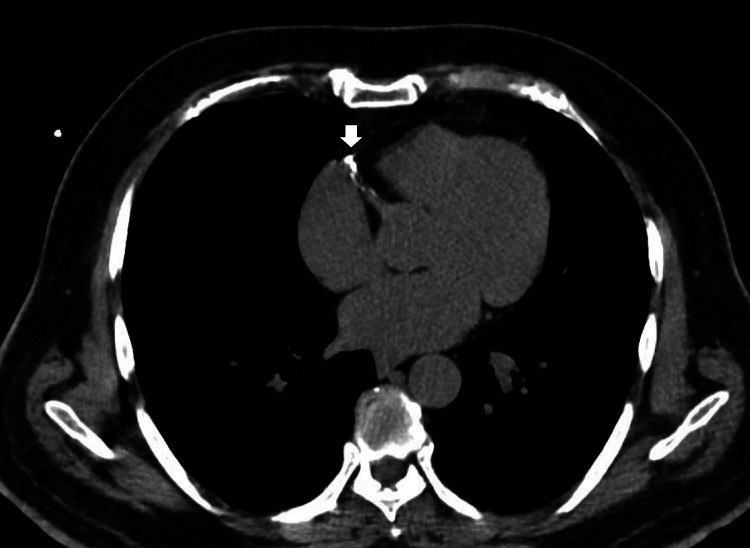
CT image of the calcified RCA The white arrow represents the lesion. RCA, right coronary artery Image source: Korosoglou et al. (2008) [12]

**Figure 4:**
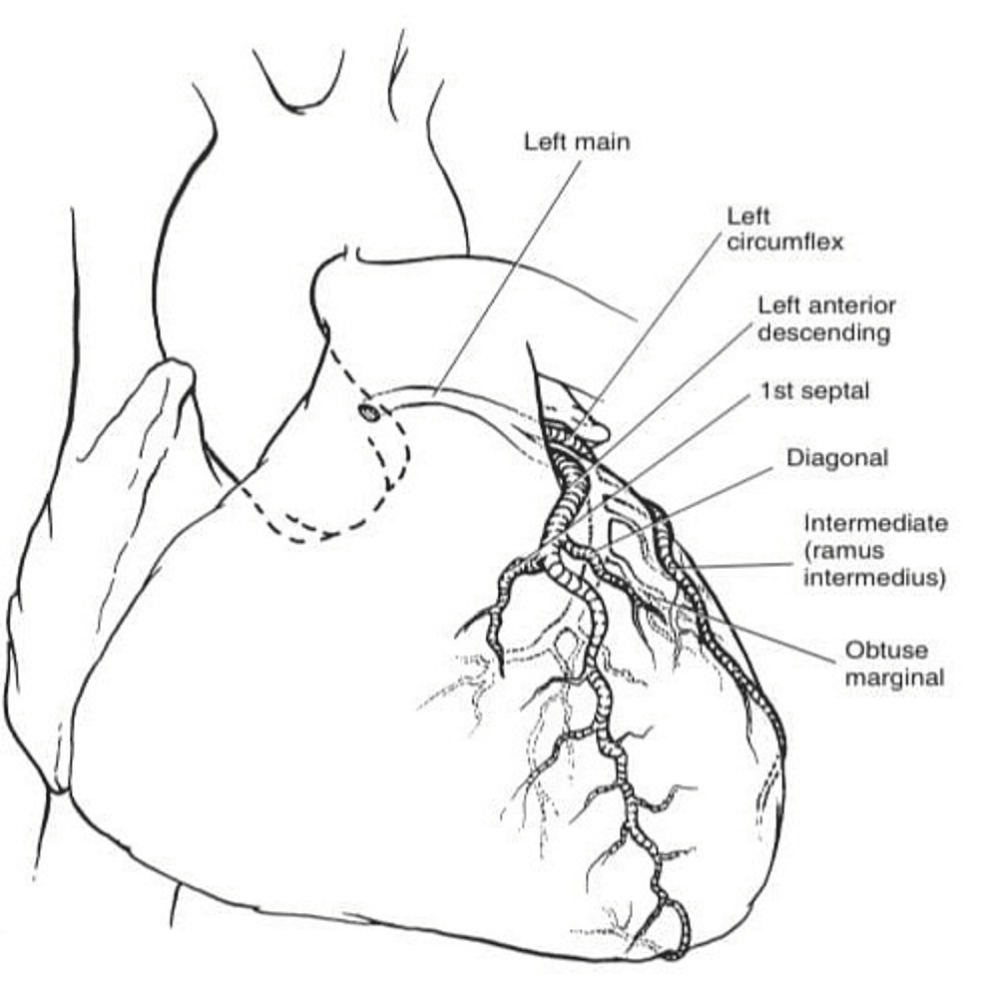
Major branches and subbranches of normal coronary arteries anatomy with LCA circulation LCA, left coronary artery Image source: Kouchoukos et al. (2013) [11]

**Figure 5:**
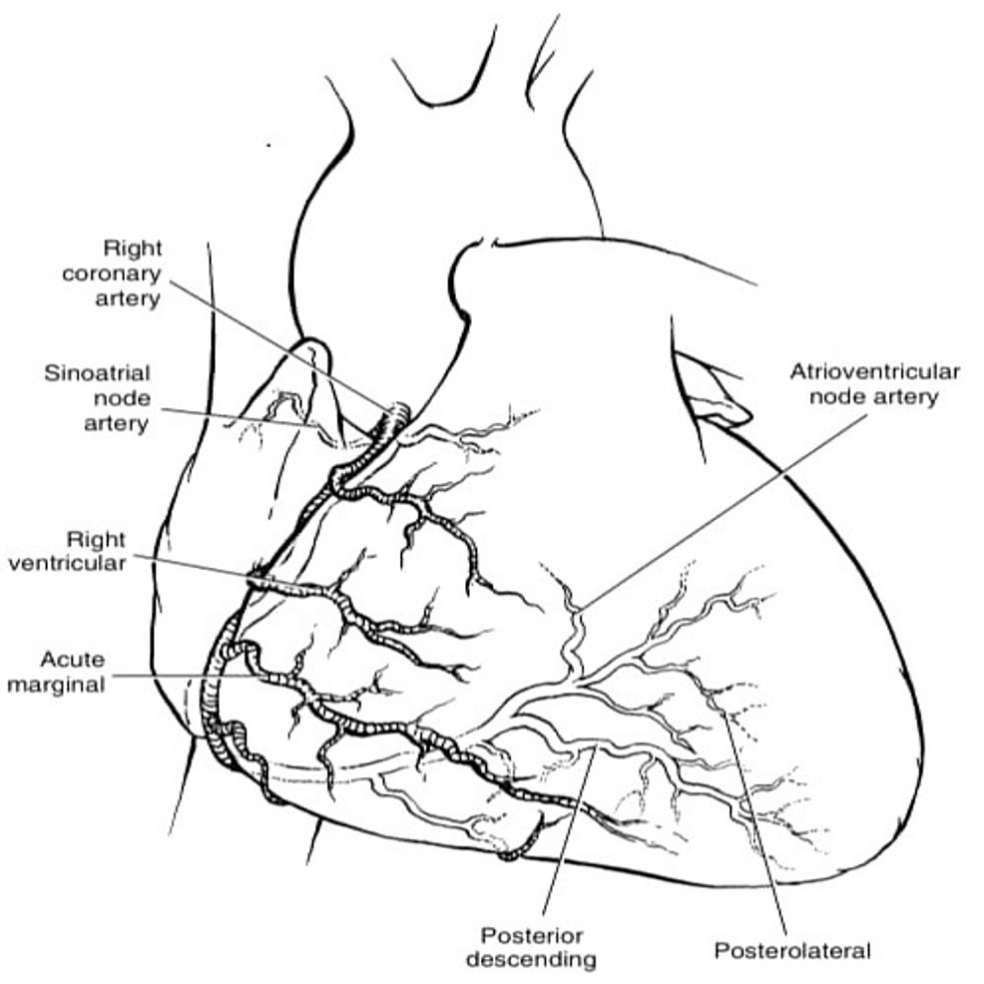
Major branches and subbranches of normal coronary arteries anatomy with RCA circulation RCA, right coronary artery Image source: Kouchoukos et al. (2013) [11]

**Figure 6:**
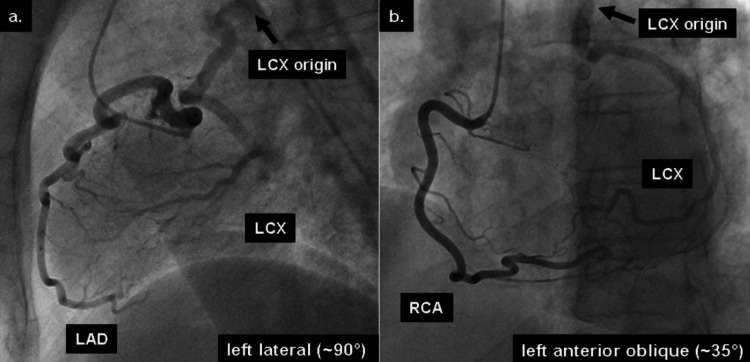
Demonstration of an anomalous origin of the LCx coronary artery from the right pulmonary artery. CA indicates the filling of the anomalous LCx coronary artery through the retrograde filling of collateral vessels following the contrast injection into (a) the LAD and (b) the RCA CA, coronary angiography; LAD, left anterior descending artery; LCx, left circumflex; RCA, right coronary artery Image source: Korosoglou et al. (2008) [12]

**Figure 7:**
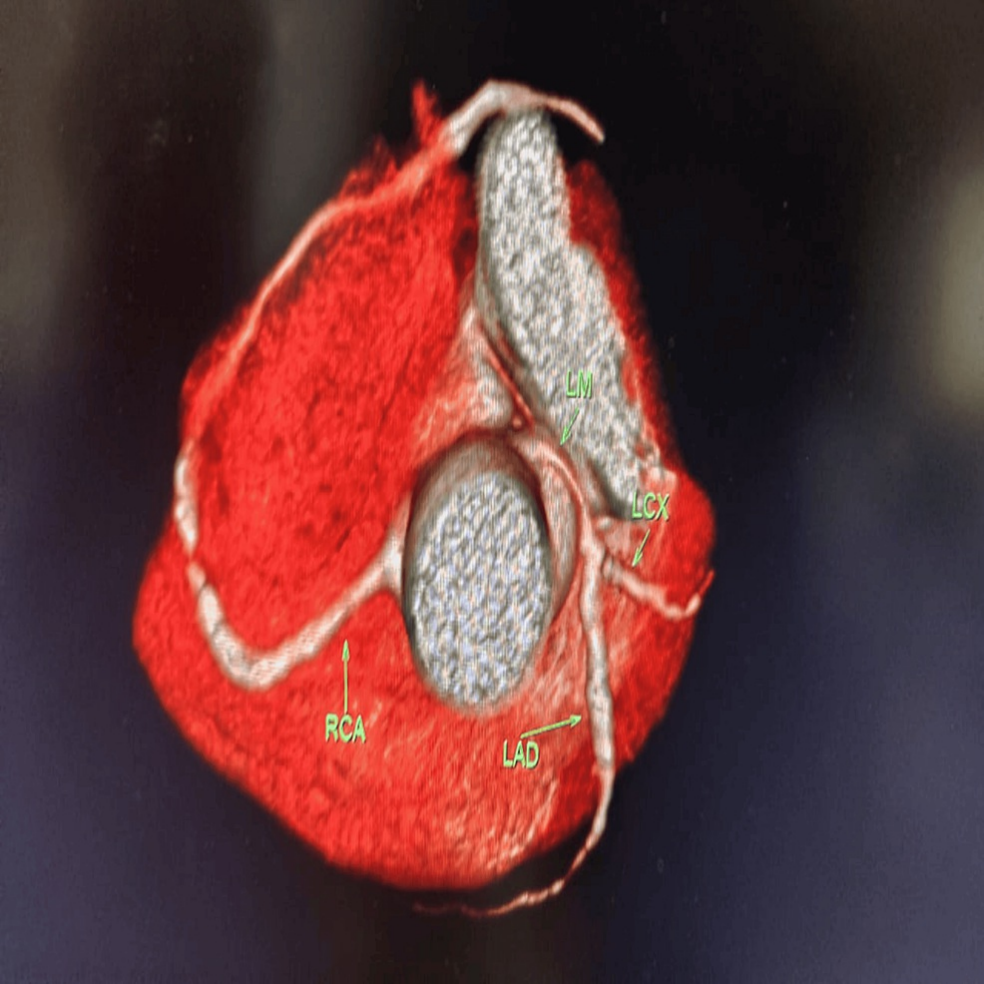
Depiction of the STJ variant origin of the LCA along with a coexisting HLCx coronary artery with SRCA circulation HLCx, hypoplasia of the left circumflex; LAD, left anterior descending artery; LCA, left coronary artery; LCx, left circumflex; LM, left main coronary artery; RCA, right coronary artery; SRCA, superdominant right coronary artery; STJ, sinotubular junction

**Figure 8:**
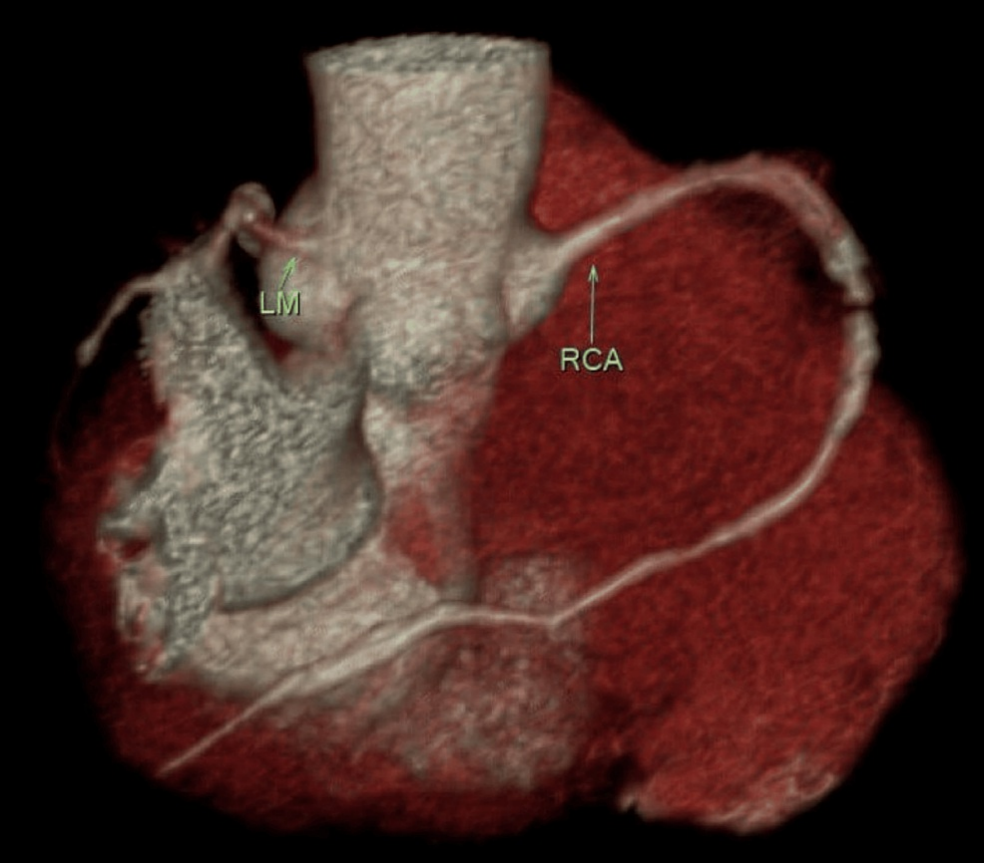
Exhibition of the ostial LM and RCA lucidly LM, left main coronary artery; RCA, right coronary artery

The subsequent CTA investigation exposed the origin of the left main coronary artery (LMCA) from the left coronary cusp, which was present at the STJ level with a retroaortic course. The LMCA caliber was found to be normal with a rough estimation of 4 mm, whereas LCx was detected to be short in course, with the proximal segment average caliber detected to be 3.5 mm, but the distal segment was established to be reduced in diameter, measured to be 1.5 mm following the obtuse marginal 1 branch, indicating HLCx. The luminal stenosis was estimated using the Society of Cardiovascular Computed Tomography. Further, the proximal segment of the LCx had multifocal eccentric calcific plaque (calcium score: 24), causing minimal stenosis (<25%) in the mid-segment (Figure 1). The left anterior descending artery (LAD), with normal caliber and type III based on its length, was detected. The LAD also had a multifocal eccentric calcific plaque (calcium score: 810) in the proximal and mid-segments, causing moderate tubular stenosis (50-69%) in the mid-segment (Figure 2). The patent posterior descending artery had an average caliber of 4.5 mm, and the large tortuous posterior left ventricular measures around 4.5 mm. The branches of the RCA provided coronary perfusion to the posterolateral and lateral walls of the cardia. Hence, RCA was determined to be SRCA in circulation. Similar to LAD, RCA also had an obstructive lesion of multifocal eccentric mixed plaques (calcium score: 1090) in the proximal and mid-segments, with moderate tubular stenosis (50-69%) in the mid-segment (Figure 3). The course and diameter of the left and right internal mammary arteries are also considered normal. On the recommendation of a CA, risk factor reduction, and routine clinical follow-up visits to the hospital, the patient was discharged from the hospital. The pericardium and cardiac valves were disease-free. Additionally, extra-cardiac structures seemed normal. A score of 3 per P4 on the Coronary Artery Disease-Reporting and Data System indicated the presence of CAD with an intermediate probability of stenosis. The images of normal coronary artery anatomy (Figure 4) of left and right coronary arteries and the anomalous origin of LCx from RCA (Figure 5) from the standard reference [11,12] were compared against the volume rendering technique image of the current reports of CAA (Figure 6) to represent the novelty of the current case report where LCA originates at the level of STJ along with HLCx and SRCA with CAD (Figure 7 and Figure 8).

## Discussion

The LMCA begins at the left aortic sinus and travels under the left atrium before separating into the LAD and the LCx after a brief distance [13]. The left and right coronary arteries, the predominant vessels that arise from the respective coronary ostium located below the STJ, appropriately ensure hemodynamic stability in individuals with a normal cardiac vascular system. An extensive CAA investigation of LCA origin has been recognized with respect to the levels of left coronary ostium ranging from 0.2 to 10 mm above, at, and below STJ in 60%, 22%, and 18% of the observed population, respectively [14]. Whereas in another examination [15], LCA configuration originated in 80% of cases, the ostium was below, and in 20% of cases, LCA origin conformations were established at the STJ level. The level of ostium was below STJ in a significant proportion of 80%, followed by 17.0% at the same level, and above it in 3.1% of cases [16]. The previous investigation also revealed the percentages of cases with the level of ostia above the STJ at 35%, at the STJ at 32%, and below the STJ at 33% [4]. There have been manifestations of abrupt deaths in young people in whom the autopsy revealed a slit-like left-sided ostial opening [17]. The case series report [18] described the LCA in one patient, which originated from the non-coronary sinus, while in another patient it originated from a mixed trunk and coursed anteriorly between the aorta and the pulmonary artery, at the level of the STJ, whereas the ascending aorta above the STJ is where the left and right coronary arteries are in the third patient. A 54-year-old Caucasian man who had stable angina and difficulty in cardiac catheterization had a CT angiography, which showed high take-off left main and right coronary arteries [19]. Hence, the stated case report determined that coronary intervention may be potentially challenging if variants in coronary arteries are significant. The intensifying utilization of diagnostic and therapeutic interventional methods has led to a recent emphasis on the importance of having extensive knowledge of the basic anatomy of LCA, where for the manipulation of catheter tips during CA, the location and level of the ostia are the critical factors. In agreement, our case report has also exhibited the CAA anomaly of coronary ostium present at the level of STJ, which is often stated as discussed above.

Hypoplastic CAD (HCAD), identified in 1970, is a rare illness characterized by a constricted luminal diameter or a shortened coronary artery course. The LMCA, proximal LAD, proximal RCA, and proximal LCx artery have normal dimensions of 4.08 mm, 3.27 mm, 3.20 mm, and 2.97 mm, respectively, as per the Indian population study on coronary artery average diameter assessment [20]. De Giorgio et al. [21] identified two autopsy occurrences with HCAD in adults and children. In the first case, a 35-year-old woman who had previously experienced exertional dyspnea had severe hypoplastic LCx with a diameter measuring less than 1.0 mm. In the second case, a nine-year-old girl died when her LCx and LAD, which measured 0.8 mm in diameter (normal: 1.8 mm), showed microscopic evidence of ischemia associated with HCAD [21-23]. Sangita et al.’s latest study in 2023 [24] included the case of a female 25-year-old who had previously experienced loss of consciousness after exertion. Her autopsy and histology examinations revealed that the lumen of all coronary arteries was constricted, with thin vessel walls and an undeveloped and absent muscle layer in the vascular walls of the LAD and LCx arteries, respectively. The infrequent congenital finding of LCx agenesis in the atrioventricular groove is commonly linked with the presence of a rare SRCA (R-I variant of the Lipton classification), which supplies the LCx territories (inferior, posterior, and lateral regions of the myocardium). Besides its own, the incidence of absent LCx has been reported to be 0.067% [25]. The past literature had not established the association of HLCx with SRCA except with absent LCx. The possible clinical feature mechanism that has already been defined is centered on coronary spasm after endothelial insult, squeezing of the myocardium, hypoplasia of vessels, etc., and the other is the “steal phenomenon” [26], in agreement with our case report since the behavior of the steal phenomenon would prevail with HLCx. CAA had not demonstrated any association with CAD pathogenesis through a literature survey [27]. In disagreement, our case report showed the association of mild to moderate CAD concerning proximal LAD, LCx, and mid-RCA with LCA origin at the level of STJ with HLCx and SRCA, but the etiological atherosclerosis mechanism behind CAA is not known. The authors probably advocate that CAD, besides the CAA, could have been mediated by the risk factors of diabetes and hypertension associated with the current case. Our case report established the occurrence of STJ variant origin of LCA along with coexisting HLCx with SRCA along with the burden of mild to moderate CAD involving proximal LAD, LCx, and mid-RCA for the first time in the literature. The clinical manifestations of the current case report medical condition, which included angina, dyspnea, and palpitation with one episodic event of syncope, are similar to those of acute coronary syndrome in that they rarely result in a large catastrophic event but are not severe [25]. The clinical features associated with our case might be attributable to any one of the two mechanisms comprising coronary spasm or coronary steal, as suggested above [26].

The present case report recommends that aberrant LCA origin at the STJ level, along with HLCx and SRCA, besides CAD encompassing LAD, LCx, and RCA, could be malignant if not treated at appropriate timing. Hence, careful consideration of anatomical variations with respect to the position of the coronary ostia, the initial parts of the coronary arteries in relation to the aorta (LCA angles of division), the coronary ostia distance from the STJ (border between the aortic sinus of Valsalva and the ascending aorta), the coronary ostia position inside the corresponding aortic sinus relative to its midline (a vertical line perpendicular to the intercommissural line of the aortic valve leaflet that divides the aortic sinus in half), and the angles between the longitudinal axis of the aorta and the initial juxtaostial parts of the arteries should be routinely practiced in patients with CAAs. Greater comprehension of the structural differences in the major blood vessels and the human heart could assist in mitigating potential risks during particular cardiosurgical interventions.

## Conclusions

The current report establishes the combination occurrence of LCA anomaly origin at the STJ level along with HLCx and SRCA conditions with the burden of mild to moderate CAD comprising proximal LAD, LCx, and mid-RCA in the literature for the first time. Moreover, the presented case carries the risk of malignancy; hence, with the advancement of modern imaging technologies, CTA should be the first choice of imaging modality rather than CA in the currently described patient to prevent fatal outcomes. Moreover, defining the coronary artery anatomy becomes critical as coronary cannulation becomes difficult in patients who require open heart surgeries. Interventional cardiologists, cardiothoracic surgeons, and radiologists must understand the anatomy of the coronary heart and whether any related anomalies exist.
